# Understanding the importance of Gender Action Planning in EC Horizon projects: a case study

**DOI:** 10.12688/openreseurope.16016.1

**Published:** 2023-09-21

**Authors:** Nicholas Fair, Daniela Kirschberger

**Affiliations:** 1 IT Innovation, University of Southampton, Southampton, England, UK; 2 Im Stadtgut Zone D1, Profactor, Steyr, 4407, Austria

**Keywords:** Gender Action; Horizon projects; Gender Equality; Gender Balance

## Abstract

Against a backdrop of continued gender challenges within the European manufacturing and technology domains, and wider society in general, such as gender balance and inequality, the gender pay gap, the glass ceiling/sticky floor, the glass cliff, the invisible woman and the under-representation of women in STEM careers and senior positions, and framed within the latest European Commission guidelines and requirements on equality, diversity and inclusion, this paper will report on the actions and research undertaken by the voluntary Gender Action Planning (GAP) team within a large, multinational, complex Industry4.0 Horizon2020 research and innovation project to try to address gender inequalities and gender balance, as well as to provide safe spaces, supportive communities and raised awareness of gender issues over the four year lifespan of the project. It is hoped that the learning from the GAP team may provide a good exemplar for future Horizon programme proposal submissions where a Gender Plan is now a mandated requirement.

## Plain language summary

This paper reports on the actions and findings of the Gender Action Planning (GAP) team within the Zero-defect Manufacturing Platform Horizon2020 project. It positions this activity within the wider context of social and workplace gender issues, as well as demonstrating how gender concerns within individual Horizon projects can be addressed. It provides a critical evaluation of the GAP team actions explaining what worked and what did not in order for future consortia to learn from, apply, improve and develop meaningful gender action plans in Horizon projects.

## Introduction

The Fourth Industrial Revolution, also known as Industry4.0 (I4.0), has been transforming the way we live and work. It involves the integration of technologies such as artificial intelligence, robotics, and the Internet of Things to create smart, interconnected systems that can analyse and respond to data in real-time. As the European Union (EU) seeks to lead the world in this new era of technology, the Horizon2020 programme, now the Horizon Europe programme, has been established to support research and innovation in this field. However, as new technological developments should now be understood as a sociotechnical phenomenon, where technologies and the societies within which they are used interact with each other to shape the development of the other (e.g. Bijker, 1997; Cartelli, 2007; Cooper & Foster, 1971; Kelly, 1978; Trist & Emery, 1960), it is vital to foreground not only technological considerations, but also the social challenges, including gender issues (e.g. Suchman, 2002), that are connected to the development of those technologies.

Against this sociotechnical backdrop, there has been a gender aspect for the past decade in European Union funded research and innovation areas, for example, as articulated in the July 2012 Communication ‘A Reinforced European Research Area Partnership for Excellence and Growth’ ( EC, 2012a); in Article 15 of the Horizon2020 research programme ( EC, 2011) ( Madariaga, 2013); and in the report 'Enhancing excellence, gender equality and efficiency in research and innovation' ( EC, 2012b). More recently the European Commission has published the Gender Equality Strategy 2020–2025 and a number of reports on the EU gender pay gap situation (e.g., European Commission, 2021). There have also been a string of national initiatives and regulations over the past two decades designed to promote gender equality in work environments, such as Gender Pay Gap reporting, 2017; Women on Boards, 2011; and Shared Parental Leave, 2015, in the UK.

The European Commission (EC) has moved the gender agenda forward with the latest Horizon Europe programme by including the explicit aim of addressing key societal challenges, as well as promoting scientific and technological excellence. To this end, funding proposals submitted to the Horizon Europe programme must, for the first time, now include a formal Gender Equality Plan (GEP), a requirement to have an institutionally-specific plan to promote gender equality in research and innovation activities. This increased focus on gender matters chimes with the increased focus on human-centered design, one of the guiding principles of resilience, sustainability, and human-centricity that defines Industry5.0 (I5.0). It also aligns with burgeoning social movements such as the Everyday Sexism Project (everydaysexism.com) and #MeToo campaign (e.g., Hansson *et al.,* 2023), gender self-identification, the adoption of non-binary identities, especially by younger people, and in the English-speaking world, new linguistic norms to account for a non-binary identity – typically changes to personal pronouns (e.g., using ‘they/them’ as opposed to ‘he/him’ or ‘she/her’).

The Industry5.0 principle of human-centered design emphasizes the importance of designing technologies and processes that are easy to use, safe, and accessible to all. It involves taking a user-centered approach to design involving understanding the needs and preferences of users, and designing technology and processes that meet those needs. Clearly, gender concerns become immediately relevant within this vision, as people of non-male gender form a significant group of users for whom software and hardware developments must be designed, but who remain underrepresented in both the design and production of such technologies. This underrepresentation of non-males in certain domains, especially Computer Science, Engineering and Manufacturing, has been recognised for many years, with large-scale programmes to support and encourage non-males into the STEM subjects (Science, Technology, Engineering, Maths), such as the Athena SWAN Charter (UK), the National Science Foundation’s ADVANCE programme (USA) and the Women in Science and Engineering programme (Canada) all running for over two decades, and the slightly more recent UN and EU initiatives such as the European Institute for Gender Equality (2010), UN Women (2010), and Women in Digital (2018) also being of significance. However, despite improvement, achieving widespread, genuine gender balance and equality in both the technology and manufacturing domains has remained illusive. For example, the WomenTechNetwork reports that in the EU just 19% of ICT jobs are held by women (2023), and within the manufacturing sector (the largest of all EU economic sectors with over 32 million workers ( statista, 2022)), women constitute roughly only 20% of the workforce ( Wyman, 2022). Furthermore, for those women who do go into STEM careers they face the ‘double trouble’ of being outnumbered and negatively stereotyped, which impacts on their career progression ( van Veelen *et al.*, 2019). The fact that despite these recent and historical initiatives, rules and regulations there continues to be a wide-ranging set of gender issues and inequalities in the workplace, under-representation of women in STEM careers, and barriers to career progression for those who do enter STEM careers, suggests that progress on these matters remains slow and incremental. Clearly action continues to need to happen.

This paper will therefore report on the activities and outcomes of the Gender Action Planning (GAP) team established within a large, four-year, Industry4.0 H2020 Innovation Action project called Zero Defects Manufacturing Platform ZDMP (ID: 825631). It will begin by positioning the GAP team as a response to some of the broader gender challenges facing women in the workplace and the specific EC Equality, Diversity and Inclusion guidelines, before detailing the activities and outcomes of the GAP team in trying to fulfil those goals and guidelines. It is hoped that by effectively positioning GAP team activities within this wider context and sharing GAP team experiences, this paper may prove a valuable resource for writing the Gender Equality Plan sections of future EC funding bids and serve as a best-practice example for project managers and work package leads once the bid has been successfully won.

## Gender challenges in the workplace

There remain a considerable range of gender challenges that are faced by individuals in the workforce, ranging from pay, to invisibility, to discrimination, to harassment. These effects can form part of an individual’s lived experience, or be evidenced in structural, systemic, or data analysis. To date the majority of research in this area has focused on gender issues within the paradigm of male and female, but of course, with the emerging occurrence of transgender and non-binary individuals these gender challenges will multiply and become even more pressing. The most widely accepted and researched adverse gender-related phenomena include:

1. *The gender pay gap*: or the disparity in earnings between men and women, where on average, women earn less than men for the same work (e.g. Perrons & Plomien, 2013; Picatoste *et al*., 2022; Plantenga & Remery, 2006; Rubery & Grimshaw, 2011), for example, the EC reported in 2021 that on average women in the EU earn 13% less per hour than men in equivalent roles ( EC, 2021).

2. *Lack of representation in leadership roles*: women are often underrepresented in leadership roles, including executive positions and on corporate boards. This is known as the "glass ceiling", whereby, despite progress in recent years, women continue to be underrepresented in leadership positions (e.g., Cheung & Halpern, 2010; Morrison *et al.*, 1987; Vinnicombe *et al.*, 2020), for example, according to a report by the European Commission, only 37% of managers, 28% of board members, 18% of senior executives and 8% of CEOs in the EU are women ( EC, 2020).

3. *Discrimination and (unconscious) bias*: women can face discrimination and bias in the workplace based on their gender, such as being passed over for employment and promotions or receiving lower performance evaluations than their male colleagues (e.g., Derks *et al.*, 2016; Heilman & Eagly, 2008; Steinpreis *et al.*, 1999).

4. *Sexual harassment and assault*: women can experience sexual harassment and assault in the workplace, which can create a hostile work environment and negatively impact mental health and job performance (e.g., Karami *et al.*, 2021; Maypole & Skaine, 1983; Minnotte & Legerski, 2019) .

5. *Lack of flexible work arrangements and maternity/family leave policies*: women are often the primary caregivers in families and may need flexible work arrangements, such as part-time or remote work, in order to balance work and family responsibilities. Equally, women may face challenges in taking maternity leave or family leave due to a lack of supportive policies or a negative culture around taking time off work (e.g., Albion, 2004; Atkinson & Hall, 2009; Chung & van der Horst, 2018).

6. *Lack of mentorship and networking opportunities*: women may have fewer opportunities for mentorship and networking, which can make it difficult for them to build relationships and advance their careers (e.g., Ehrich, 1994; Harris, 2022; Orser *et al.*, 2012; Wang, 2009).

7. *Stereotypes and microaggressions*: women may face stereotypes and microaggressions in the workplace, such as being perceived as less competent or being subjected to gendered comments or jokes (e.g., Nadal *et al.*, 2021; Sue, 2010; Sue & Spanierman, 2020.

These work-related gender issues are also accompanied by gender issues in research and design. In Caroline Criado Perez's book, "Invisible Women: Exposing Data Bias in a World Designed for Men," (2019) the author highlights the pervasive phenomenon of the "invisible woman" in our society. The argument is that data and research are often biased towards men and fail to take into account the experiences and needs of women, resulting in a world that is designed primarily by men for men. This can have serious consequences for women, who may face discrimination, health risks, and other negative outcomes as a result.

Perez cites a range of examples, from the design of medical equipment that does not take into account the differences in women's bodies, to the fact that women are more likely to be injured or killed in car accidents due to the fact that crash test dummies are modelled on male bodies. She also highlights the fact that women's contributions to history, science, and other fields are often overlooked or erased, perpetuating the idea that women are "invisible" or less important. Overall, the Invisible Woman phenomenon highlights the need for greater attention to gender bias and inequality in research, policy, and design.

Alongside the Invisible Woman phenomenon are other equally problematic gender phenomena including the corollary to the Glass Ceiling described above – the Sticky Floor – which highlights the tendency of women to remain stuck in low-wage jobs with limited opportunities for upward mobility despite having the necessary skills and qualifications (e.g., Harlan & White Berheide, 1994; Morgan, 2017). This phenomenon reflects the gender issues outlined above in that there are several factors that contribute to the Sticky Floor phenomenon, including stereotype bias, societal expectations, and caregiver responsibilities. Women are often stereotyped as being less capable or committed to their careers than men, which can lead to discrimination in hiring and promotion decisions. Additionally, women are expected to fulfil traditional caregiving roles, such as raising children and caring for elderly parents, which can limit their availability for work and make it difficult to pursue higher-paying jobs. The consequences of the Sticky Floor are significant, as it perpetuates gender inequality and perpetuates the gender wage gap, while women who are trapped in low-wage jobs have limited economic opportunities and are more likely to experience financial insecurity and poverty.

A final gender-related phenomenon is the Glass Cliff Edge (e.g., Morgenroth *et al.*, 2020; Ryan *et al.*, 2007; Ryan *et al.*, 2016), which refers to the phenomenon where women are more likely to be appointed to workplace leadership positions in times of crisis, when the risk of failure is high. This can be seen as a form of tokenism, where women are brought in to solve problems that have been created by men and may often result in women leaving the role or even the workforce altogether as a result of stress, a lack of support and resources, and a sense of being set up to fail.

## Gender within European Commission Horizon research programmes

Clearly it is beyond the scope of a single EC Horizon project (either Horizon2020 or Horizon Europe) to address the gender issues outlined above on a society-wide basis, however, it is possible for action to be taken within the limited parameters of the project to try to minimise the impact of these issues and to create a gender equal project working environment. Within the Commission itself the Equality Diversity and Inclusion (EDI) Guidelines, published in October 2018 and covering all EU institutions and agencies in respect of recruitment, training and development, workplace culture, and the provision of support and resources, provides a set of useful gender-related concepts that should be fully considered from the outset in order to promote gender equality in a Horizon project.

Firstly, this includes incorporating gender perspectives into all aspects of the project, including research design, data collection, analysis, and reporting. Known as Gender Mainstreaming, this helps to ensure that the project is sensitive to the needs and experiences of both men and women and goes some way to addressing the Invisible Woman problem. Secondly, there should be a commitment to Gender Balance whereby both men and women are represented in all aspects of the project, including leadership positions, research teams, and advisory committees. This helps to ensure that the project benefits from a diversity of perspectives and experiences and helps stimulate innovation, as well as helping to address the under-representation of women in leadership roles. Next is the requirement for Gender-sensitive Communication, where all language and images used in project deliverables, meetings, plenaries and presentations are inclusive and non-discriminatory, and avoid reinforcing gender stereotypes. This helps to create a culture of respect and inclusivity within the project and raises awareness of the importance of language in shaping an individual’s daily workplace interactions, with the potential outcome of reducing the occurrence of microaggressions, such as inappropriate or offensive jokes for example. A final important aspect of the Guidelines is the notion of Gender-sensitive Training, which has two elements. Firstly, it involves providing training and professional development opportunities that are accessible to both men and women, and that take into account the different needs and experiences of each group. This helps to ensure that everyone has the skills and knowledge necessary to participate fully in the project. Secondly, it can also refer to training provided to project team members that is designed to explicitly educate around gender issues themselves, which can serve to improve the understanding of gender issues across the project team and encourage a commitment to maximising gender equality and respect.

The EC’s EDI Guidelines also recommend a number of good practices for achieving better gender equality in the workplace, including regularly monitoring and evaluating the project's progress towards achieving gender equality, and making adjustments as necessary; providing support and resources to women who may face barriers to participation in the project, such as childcare or flexible working arrangements; encouraging women to take on leadership roles within the project, and providing them with the necessary support and resources to succeed; and creating a culture of respect and inclusivity within the project, where all members feel valued and supported. Therefore, framed within the macro, social domain of on-going societal gander issues, and guided by the advice of the EC’s EDI Guidelines, this paper will now turn to the specific gender actions implemented in Horizon2020 Zero-defects Manufacturing Platform (ZDMP) project as a case study exploring how these ideas and ideals can be enacted in large-scale, multinational, highly-complex, innovation projects.

## Gender Action Within a Horizon2020 Project: A Case Study

The Horizon2020 Zero-defects Manufacturing Platform (ZDMP) project is a large-scale innovation action funded by the European Commission under Grant Agreement 825631 running from January 2019 to June 2023. It involves 31 partners (Manufacturers, Technology Providers, Consultants and Research Institutes) from 11 countries with a total budget of circa 16.2M€, with a focus on developing and providing an extendable platform and smart, AI-driven tools and services, including a I4.0 marketplace, to support factories with a high interoperability level in achieving the goal of zero-defect production.

In total 156 individuals worked extensively on the ZDMP project for its duration (additional people were involved at various points in the four-year project, but not throughout), of which just 30 were women (19%). This closely mirrors the percentage of women in the wider EU manufacturing workforce (20%) ( Wyman, 2022), and reinforces the wider concerns of female under-representation in STEM careers. In addition, the project consisted of 13 work packages, 3 of which were led by women (23%), which slightly exceeds the EU average of 18% of senior executives being female ( EC, 2020). Finally, the ZDMP Advisory Board consisted of four people, one of whom was female (25%), which also is in general alignment with EU female Board membership averages (28%) (ibid.). In other words, the ZDMP project can be reasonably considered as ‘typical’ for a workplace in the EU manufacturing and technology context and is therefore a useful case to study.

Despite this, it was also clear from the outset that females constituted a minority and were ‘outnumbered’ four to one by males. Consequently, in Year 1 of the ZDMP project, with the approval of the Project Co-ordinator and Manager, a voluntary Gender Action Planning team (GAP team) was set up consisting of equal numbers of males and females (3 of each gender from 5 different countries) with responsibility for planning gender equality actions and monitoring and reporting on gender equality progress. Immediately the voluntary nature of this team highlighted a systemic issue within Horizon projects, namely that gender action planning, monitoring and reporting is not normally budgeted for in the initial funding application nor in the final Grant Agreement, which quite literally devalues gender equality concerns when compared with other forms of project activity.

Nevertheless, in Year 1 the GAP team undertook a series of planning actions aimed at addressing some of the potential areas of gender inequality (as detailed above in this paper). Firstly, in response to the suggestion for Gender-sensitive Communication the GAP team planned to review all project deliverables and all spoken interactions (e.g., team meetings, plenaries, presentations, *etc*.) to ensure gender-neutral language and imagery was used throughout. This was implemented during Years 2 to 4 of the project with considerable success. For example, in the first half of the project there was a natural, historical tendency for the more experienced male project members to refer to the effort allocation for tasks in terms of ‘man months’. This phraseology was repeatedly seen in written documents and heard verbalised in meetings and presentations. Through continued and repeated direct interventions by the GAP team over two years the use of ‘man months’ was gradually superseded with the term ‘person months’ in line with modern EC norms. By the end of the project all verbal and written communications used ‘person months’ by default and out of habit and ‘man months’ had become a forgotten term.

On the other hand, it is worth noting that a second very common everyday gendered phraseology was not successfully changed, namely the use of ‘guys’ to refer to everyone (as in the phrase ‘Hello guys’ or ‘Right guys lets now talk about…’). In fact, the GAP team efforts to point out that for the women in the project this felt like gendered language and left them feeling excluded was actively rejected by many males, who claimed that it could refer to both males and females and was not gendered at all. Lively public debates on this matter during project plenaries led to formal dictionary definitions being called up, which supported both sides of the argument (US English definitions referring to both genders, while UK English definitions referring to males), and no agreement was reached on the appropriateness of ‘guys’ in verbal communication. This was actually disappointing, as the fact that the female project members had expressed the feeling that this was exclusive of them and that they did not like it should have been enough on its own to drive a change in language use, without any recourse to dictionaries, formal definitions or debates. The fact that it wasn’t enough further indicates that when it comes to respecting Gender-sensitive Communication many males still have some considerable distance to travel. Nevertheless, the continuous public monitoring and ‘calling out’ of gendered language by the GAP team did serve to significantly raise awareness in the minds of all members of the ZDMP project of the importance of language in creating an equal and inclusive working environment, even if change was not always forthcoming.

Secondly, in response to a lack of support and mentorship for women, the GAP team established a women-only mailing list in Year 1. All women in the project were invited to join the mailing list and roughly once a quarter informative emails concerning funding opportunities for women, interesting news or academic articles, or helpful work tips were sent to the group by the GAP team (10 in total). The mailing list also provided a safe space within which women could communicate, support each other and share knowledge, experience and best practice. Although this proved unnecessary (thankfully!) it was also a channel to help combat sexual harassment and/or everyday sexism as women could, if required, warn each other of inappropriate behaviour by individuals and support each other in taking any necessary formal actions resulting from harassment incidents.

Next, in order to (gender) balance the women-only mailing list the GAP team also set up a free suggestion box for all project members. This was used for anonymous complaints about any incident or areas of concern for project members on gender and other issues, including discrimination, a lack of inclusivity, and any other workplace problems, as well as for making positive suggestions for improvements in these areas. The suggestion box was monitored and managed by the GAP Team, and submitted suggestions were discussed by the team during the monthly team meetings, where the appropriate actions to be taken were explored and agreed. Where required the suggestions raised were brought to the attention of the Project Manager and the senior project management team, who would deliberate further on the issue before agreeing and implementing any necessary actions. This provided a safe channel for all project members to raise concerns that they may otherwise have felt uncomfortable doing in more public forums.

Finally, the female members of the GAP team also each wrote a two-page blog post outlining their career progression, detailing any obstacles or challenges that had been faced as a result of their gender, and providing guidance on how those were overcome. These blogs were published on the ZDMP website in full and without editing or interference from the ZDMP senior management team. This was designed to enable the sharing of lived experience and acted as a guide for other women within the project who were at earlier career stages or in more junior positions. Alongside this, the GAP team was also provided with regular thirty-minute presentation slots on the agenda at the quarterly ZDMP project plenaries, where general issues could be raised and the GAP team actions, progress and monitoring could be reported to the whole project team. For this to happen the on-going positive support of the Project Co-ordinator and Manager and senior management team was required and received, as they were responsible for drawing up the agendas. However, on a number of occasions, where other plenary agenda items overran, or were considered of such urgency that they needed longer presentation/discussion time, it was most often the GAP team gender session that was either cancelled or moved to later time slots or days. Again, this was a very visible indication of the lack of priority and importance assigned to gender issues in comparison with other aspects of the project (such as technical development, exploitation, dissemination, cascade funding…etc.). Nevertheless, the fact that the GAP team did get to present at many plenaries was far better than not presenting at all and did contribute to raising and maintaining awareness of gender issues across the project members. Overall, these actions helped to create a sense of community for the women in ZDMP, provided supportive and safe spaces and channels for self-expression, provided awareness-raising opportunities, and minimised as much as possible the casual everyday use of unacceptable gendered language.

In an effort to address Gender Balance, the GAP team also worked closely with the lead for the Cascade Funding (Open Calls) work package to try to ensure a gender balance in the Call Evaluation team and in the sub-project mentoring team. During the ZDMP project two Open Calls were held, distributing €3.2million to SMEs across Europe. The target was to achieve a 50/50 gender balance in the evaluation team in order to ensure the best level of evaluation possible. However, despite concerted efforts during evaluator recruitment, just 22% of external evaluators and 20% of internal evaluators were women, meaning a 21/79 split in favour of males. Although this aligns with the general lack of gender balance within the manufacturing and technology domains ( Wyman, 2022) it was nevertheless disappointing and serves to highlight the broader issues regarding women in STEM careers. Similarly, when it came to assigning mentors to the successful winning projects from the Open Calls from within the ZDMP project team, and despite direct appeals to the women of ZDMP via the GAP team, only 3 of the 28 subprojects were mentored by women (11%). Many women cited a lack of experience and/or a lack of confidence when turning down the opportunity to become a subproject mentor. Indeed, one of the three women who did take on the mentoring role only agreed to do so if the work package lead would agree to informally and voluntarily mentor and support her throughout the process (which of course was agreed to immediately). This further indicates the systemic lack of formal mentorship opportunities and mentoring funding provision for women in Horizon projects, and the difficulties in achieving a 50/50 gender balance in the manufacturing and technology domains.

In addition to the practical actions implemented by the GAP team so far outlined, it was also decided that conducting some primary research into gender issues, the lived experience, and the impact of the GAP team was of value. Hence, the GAP team developed and published a Gender Survey, which was released firstly in Month 18 of the project and then repeated in Month 34 in order to establish a baseline (M18) and then make an assessment of the impact of the GAP team actions (M34) (see “Extended Data” Fair, 2023). In total 123 responses were received (71 in survey 1 and 52 in survey 2), with 34 individuals completing both surveys, of which 25 responses were from females (20%) and 98 responses from males (80%), and 70 responses from individuals in non-leadership roles (57%) and 53 from those with leadership responsibilities (43%).

Firstly, just over three quarters of all respondents who had previously worked on EC Horizon projects reported that this was the first time that Gender Balance and Inequality had been raised within a project and meaningful gender actions implemented, which indicates the low priority given to gender matters in Horizon projects generally. Turning to the ZDMP project specifically, in the M18 survey 31% of female respondents reported feeling intimidated by the speech or actions of ZDMP colleagues, compared with 8% of males reporting the same, with the language used in emails being rude, inappropriate, sarcastic, demotivating, unprofessional and/or demeaning being cited as the main problem. In the M34 survey, no one (of either gender) reported feeling intimidated by the speech or actions of others. Secondly, in the M18 survey 19% of female respondents reported feeling discriminated against because of their gender and 31% felt like they had not been taken seriously in their work as a result of their gender (only 2% of males reported feeling discriminated against and no males felt that they had not been taken seriously). Again though, in the M34 survey this had reduced to zero for both genders in both aspects. Similarly, with reference to stereotypes and microaggressions, 4% of female respondents in the M18 survey reported feeling uncomfortable during physical meetings and plenaries resulting from behaviours such as being called ‘baby’, being touched on the hands or shoulder, or having their dress/clothing publicly referred to by male presenters or colleagues. In the M34 survey this had also reduced to zero. Overall, the change from the M18 situation to the M34 situation indicates a positive GAP team impact on respectful everyday workplace communication, gender discrimination and stereotyping/microaggressions.

It is also interesting to note that the M18 survey indicated that where individuals had felt intimidated, discriminated against or uncomfortable, in almost half of the cases they took no action to address it (47% of occasions), citing a lack of confidence to speak up or that low-level microaggressions, especially in the form of sexist or otherwise inappropriate ‘jokes’, were difficult to confront when the majority of others were laughing along. Just 5% of respondents reported challenging gender biased or inappropriate language or behaviour at the time it occurred. A further 10% formally reported an incident through the proper channels. The remaining 38% reported talking informally with other colleagues after the event was over. This indicates the importance and value of the women-only email list and the anonymous suggestion box established by the GAP team as a safe route to report incidents and seek support, and as a way to raise issues to more formal levels without exposing oneself by doing so alone. In this regard, it is concerning that only 61% of respondents (both surveys) felt that it was Very Important to have clear, well-defined and safe processes for individuals to report inequality, discrimination or intimidation and open and transparent systems of redress should such issues occur.

This finding was the start of a concerning pattern visible in the survey data relating to broader gender issues. When asked in the M18 survey whether the respondent considered gender balanced teams ‘beneficial to creating better and more successful project outcomes’ only 56% agreed, with just 50% of the female respondents agreeing. This actually fell by the M34 survey, where just 44% of respondents agreed that gender balanced teams were better for outcomes, indicating that although some progress on the more immediate gender issues can be made, there remains an attitude that reporting channels and gender balanced teams are not really that important.

## Conclusion

The ZDMP project has been successful in implementing an on-going, concerted range of practical actions and research resulting from the voluntary efforts of the six members of the Gender Action Planning team, from which it is hoped that future Horizon projects can learn, and in their turn develop and improve. Overall the GAP team was successful in introducing improvements to Gender-sensitive Communication; the provision of safe female support structures and community building; reducing the occurrence of discrimination, intimidation, stereotyping and microaggressions; and raising awareness of gender concerns (which even led to individual partner organisations updating or extending their own existing gender inequality policies, or project members bringing GAP team actions to other Horizon projects on which they also worked). However, there remains considerable progress to be made in shifting attitudes to the value of gender balanced teams; to the importance and value of safe, supportive reporting and redress channels; to the need for systemic change in effort allocation to provide for meaningful gender action, especially in providing mentoring opportunities for women; and to the overall balance of women in leadership roles and in manufacturing and STEM careers in general.

## Data Availability

The data underlying the results cannot be shared for participant confidentiality reasons, as approved by the ethical review board on 03/07/23. For this particular publication, any additional information on data that is not retrievable online could be requested by contacting the researcher by E-Mail: N.S.Fair@Soton.ac.uk, stating the purposes of their request. University of Southampton PURE: “Dataset supporting the publication ‘Understanding the Importance of Gender Action Planning in EC Horizon Projects: A Case Study.’” https://doi.org/10.5258/SOTON/D2619 ( Fair, 2023). This project contains the following extended data: ‘ZDMP_Survey_1_2_Questions.xlsx’ (The questions asked of participants in the study) Data are available under the terms of the Creative Commons Attribution 4.0 International (CC BY 4.0)
