# Supplementary material for: Understanding the importance of Gender Action Planning in EC Horizon projects: a case study
Source: Open Res Eur. 2023 Sep 21;3:159. [Version 1] doi: 10.12688/openreseurope.16016.1 (PMC10632589; doi:10.12688/openreseurope.16016.1)
Supplement: Peer review history

# Reviewer report: Reviewer response for version 1

**DOI:** 10.21956/openreseurope.17294.r35526

**Published:** 2023-11-08

**Authors:** Sara Clavero

**Affiliations:** 1 Directorate for Equality, Diversity and Inclusion, Technological University Dublin, Dublin, Leinster, Ireland

The paper provides very useful guidance on how to integrate a gender equality perspective in EU funded projects through the presentation and analysis of a specific case-study. It will be particularly valuable source to consortia in the process of drafting an application to Horizon Europe calls. The case-study points to main obstacles encountered in implementing a gender equality action plan and how these were navigated, while also drawing attention to key challenges in progressing towards gender equality in this type of projects. 

 The paper provides a clear background and the description of the steps taken by the GAP is rich in detail. 

 I was quite impressed by the effort of the GAP team of this H2020 project and the lessons learnt from their experience will no doubt contribute greatly towards better practice in this area. 

Is the case presented with sufficient detail to be useful for teaching or other practitioners?

Yes

Is the work clearly and accurately presented and does it cite the current literature?

Yes

If applicable, is the statistical analysis and its interpretation appropriate?

Yes

Are all the source data underlying the results available to ensure full reproducibility?

Yes

Are the conclusions drawn adequately supported by the results?

Yes

Is the background of the case’s history and progression described in sufficient detail?

Yes

Reviewer Expertise:

Gender equality and intersectionality, gender and politics, gender in research.

I confirm that I have read this submission and believe that I have an appropriate level of expertise to confirm that it is of an acceptable scientific standard.

# Reviewer report: Reviewer response for version 1

**DOI:** 10.21956/openreseurope.17294.r35529

**Published:** 2023-10-12

**Authors:** Karen Johnston

**Affiliations:** 1 School of Organisations, Systems and People, University of Portsmouth, Portsmouth, England, UK

The authors provide a good case and narrative for the under-representation of women in I4.0 and I5.0. The literature is well presented and covers the main challenges and inequalities for women in the workplace and in STEMM professions, in particular. The literature I would have expected to be cited such as Eagly, Rubery, Vinnecombe and Wyman is cited in the article. The authors provide a good and clear justification for the project's GAP approach, drawing upon European Commission policies (e.g. Gender Sensitive Communication). There is sufficient information about the project, and the GAP team, approach and implementation of actions to understand the background of the case.

 The results are illuminating and honest. While there noteworthy achievements (noted with the surveys in M18 and M34), the authors also observed shortcomings (e.g. issues of prioritization). The case presents a level of empiricism as an evidence base of what works and what does not within the project GAP approach. The statistical analysis is descriptive but understandable. The conclusions that are therefore drawn are appropriate. The case would be of interest to a teaching and practitioner audience as it demonstrates better practices, but also cautions on shortcomings of GAPs in the case of this project.

Is the case presented with sufficient detail to be useful for teaching or other practitioners?

Yes

Is the work clearly and accurately presented and does it cite the current literature?

Yes

If applicable, is the statistical analysis and its interpretation appropriate?

Yes

Are all the source data underlying the results available to ensure full reproducibility?

Not applicable

Are the conclusions drawn adequately supported by the results?

Yes

Is the background of the case’s history and progression described in sufficient detail?

Yes

Reviewer Expertise:

Gender studies, public administration

I confirm that I have read this submission and believe that I have an appropriate level of expertise to confirm that it is of an acceptable scientific standard.
